# Temporal Variability of Urinary Organophosphate Flame Retardant Metabolites Among E-Waste Workers in France: A Pilot Study to Inform Occupational Biomonitoring Sampling Design

**DOI:** 10.3390/toxics14090778

**Published:** 2026-08-31

**Authors:** Baninia Habchi, Guillaume Antoine, Aurélie Martin Remy, François Zimmermann, Marie-Thérèse Lecler, Williams Esteve, Claude Emond, Nathalie Grova, Sophie Ndaw

**Affiliations:** 1 Department of Toxicology and Biomonitoring, INRS (French National Research and Safety Institute for the Prevention of Occupational Accidents and Diseases), CS 60027, 54519 Vandoeuvre-lès-Nancy, France; guillaume.antoine@inrs.fr (G.A.); aurelie.remy@inrs.fr (A.M.R.); nathalie.grova@inrs.fr (N.G.); sophie.ndaw@inrs.fr (S.N.); 2 Department of Process Engineering, INRS (French National Research and Safety Institute for the Prevention of Occupational Accidents and Diseases), CS 60027, 54519 Vandoeuvre-lès-Nancy, France; francois.zimmermann@inrs.fr (F.Z.); marie-therese.lecler@inrs.fr (M.-T.L.); 3 Department of Pollutants Metrology, INRS (French National Research and Safety Institute for the Prevention of Occupational Accidents and Diseases), CS 60027, 54519 Vandoeuvre-lès-Nancy, France; williams.esteve@inrs.fr; 4 Department of Occupational and Environmental Health, School of Public Health, Université de Montréal, Montréal, QC H3N 1X9, Canada; claude.emond@umontreal.ca; 5 PKSH Inc., Crabtree, QC J0K 1B0, Canada

**Keywords:** occupational exposure, e-waste, organophosphate esters, flame retardant, temporal variability, metabolites

## Abstract

Workers in the e-waste industry are exposed to chemical hazards, including organophosphate esters (OPEs) used as flame retardants (FRs). Assessing exposure through spot urine metabolite analysis is challenging due to limited knowledge of the toxicokinetics and urinary excretion patterns of OPEs. This pilot study investigates the temporal variability of seven organophosphate flame retardants (OPFRs) in urine samples from e-waste workers over five workdays to identify the most representative spot urine sample for accurately assessing occupational exposure. Six exposed workers provided urine samples from the first shift of the working week to the morning after the last workday. Seven metabolites, (bis(2-chloroethyl) phosphate (BCEP), diphenyl phosphate (DPHP), di-o-cresyl phosphate (DoCP), di-m-cresyl phosphate (DmCP), di-p-cresyl phosphate (DpCP), bis(1,3-dichloro-2-propyl) phosphate (BDCPP) and bis-(1-chloro-2-propyl) phosphate (BCPP)), were analyzed using liquid chromatography-mass spectrometry. DmCP + DpCP showed weekly increases and fair to good intraclass correlation coefficient (ICC), indicating moderate temporal variability across days. Conversely, DPHP and BCEP showed rapid decreases and lower ICC, suggesting higher intra-day variability. A grouping approach based on collection time and workday showed that the suitable spot urine is 2–5 h delayed post-shift for BCEP and DPHP, whereas the end of the workweek is the most appropriate time for DmCP + DpCP. This pilot study provides preliminary information on temporal variability to inform sampling design. This approach could be extended to a broader range of FRs, thereby enhancing the efficiency and accuracy of occupational biomonitoring.

## 1. Introduction

Approximately 2.3 million tons of organophosphate esters (OPEs) are used as flame retardants (FRs) every year worldwide to meet fire-safety standards and regulatory requirements across industries [1,2,3]. OPEs are widely used in various materials such as plastics, electrical and electronic equipment (EEE), wires and cables, building materials, furniture, textiles and transportation equipment, to inhibit or suppress the combustion process through chemical and/or physical mechanisms [4]. In some applications, OPEs constitute up to 30% of the composition of materials. In fact, the global FR market was valued at over $9 billion in 2023, and is expected to reach approximately $18 billion by 2033, with an annual growth rate of over 7% [5]. The European WEEE (Waste Electrical and Electronic Equipment) directive requires a 65% recycling rate for EEE, while the French Circular Economy Law sets an ambitious target of 100% of recycling for plastics. However, several FRs, particularly halogenated FRs, have been associated with persistence, bioaccumulation and adverse health effects, including endocrine disruption, reproductive and developmental toxicity, immunotoxicity, neurotoxicity [6] and cancer risk [7,8].

Regulatory trends are therefore increasingly promoting the replacement of halogenated OPEs with safer alternatives, driven by environmental and health considerations. Some of these OPEs, have already been banned in the EU Directives [1], particularly brominated flame retardants (BFRs). Consequently, an increasing number of organophosphate flame retardants (OPFRs) have emerged as substitutes for BFRs in a wide range of products. Concerns have been raised regarding their potential toxicity, and their use in the EEE industry remains widespread, with WEEE recycling representing a major contributor to their environmental dissemination. Due to their ubiquitous presence in the environment and their emissions into the air during WEEE processing, OPFRs can enter the body through inhalation, ingestion and dermal adsorption. They may therefore represent a major occupational health concern, highlighting the urgent need to investigate their exposure levels and their potential impact on workers’ health.

After absorption, OPFRs undergo Phase I and II metabolisms before being excreted, with metabolic clearance varying depending on compounds and species [9,10]. Differences between species (rats, fish, and humans) raise concerns about applying animal data to humans, even when corrected using allometric scaling. To our knowledge, only one physiologically based pharmacokinetic (PBPK) model has been developed to describe the distribution of tris(1,3-dichloro-2-propyl) phosphate (TDCPP), tris(1-chloro-2-propyl) phosphate (TCPP), and tris(2-chloroethyl) phosphate (TCEP) in rats [11]. The authors point out, however, that further research is required to determine whether these chemicals exhibit similar kinetic behavior in humans. Overall, knowledge of human kinetic parameters, including biological half-life, remains limited [10]. Furthermore, it is essential to obtain a clear understanding of the half-lives of OPFRs, which are generally considered to be short to moderate (from less than 2 h to 1 day). Recent findings suggest longer half-lives for chlorinated OPFRs, due to low hepatic and renal clearance (3 to 53 days) [10]. Given these toxicokinetic characteristics and the ubiquitous presence of OPFRs, particularly in domestic environments, urinary measurements reflect both occupational and non-occupational sources. For accurate occupational and environmental exposure assessment, 24-h pooled urine sampling is ideal for (i) providing a reliable estimate of daily exposure and reducing the risk of underestimating exposure levels, compared with single spot sampling; and (ii) improving the overall reliability of OPFR exposure assessment. However, the systematic collection of 24-h urine samples is not feasible in occupational settings; which is why spot-urine samples are commonly used in occupational biomonitoring studies despite fluctuations in exposure levels and between days; as well as limited knowledge regarding the half-lives and temporal variability of OPFRs which can potentially lead to a potential underestimation of exposure levels [12,13,14]. To optimize the use of spot urine samples, it is essential to investigate the temporal variability of OPFR biomarkers and to characterize their excretion profiles throughout the workday and workweek. Furthermore, identifying exposure-related excretion peaks and understanding intra-day variability can improve the interpretation of spot-urine measurements and enhance the accuracy of occupational exposure assessment. Previous studies have investigated the temporal variability of OPFR metabolite concentrations in the same general-population individuals by comparing spot urine samples collected during a day and/or over several days [12,14,15,16,17,18,19]. To our knowledge, none of these studies have however specifically examined the temporal variability of OPFR biomarkers in the workplace.

All of the spot urine samples were thus collected directly from workers in the WEEE sector over a one-week working period to establish an approach for assessing the temporal variability of the urinary excretion of OPFRs. The selection of the seven OPFRs investigated in this study was based on a previous systematic review focusing on human exposure to halogenated OPFRs in e-waste environments [20]. This review identified the most frequently detected OPFRs and revealed that the majority of existing studies have been conducted in China, highlighting the critical need for broader geographic representation in future research [20]. This study is therefore aimed at identifying, for each metabolite, the most representative spot urine sample for the accurate assessment of occupational exposure over a working week.

## 2. Materials and Methods

### 2.1. Chemicals

**Figure 1:**
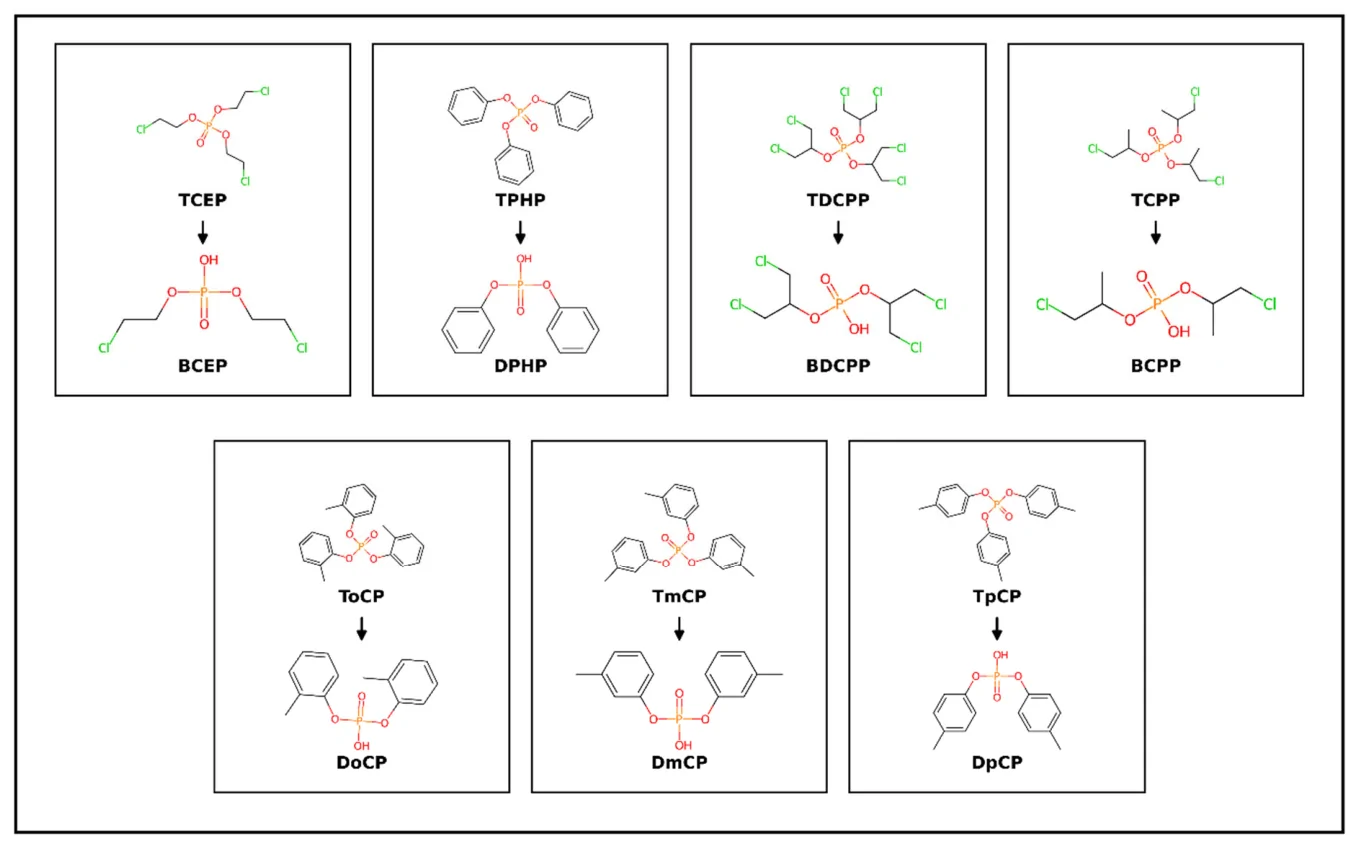
Chemical structures of the parent OPFRs and their corresponding metabolites investigated in this study. Chemical structures were generated from SMILES representations using RDKit. Arrows indicate the metabolic biotransformation of parent OPFRs into their corresponding metabolites.

Due to the low hepatic clearance of certain OPFRs such as TCEP, which may result in the urinary excretion of unchanged parent compounds, and to the limited knowledge about their metabolic pathways, both the parent compounds and the metabolites were included as target analytes in the urine analysis method [21]. Seven parent OPFRs were considered: TCEP, triphenyl phosphate (TPHP), tri-o-cresyl phosphate (ToCP), tri-m-cresyl phosphate (TmCP), tri-p-cresyl phosphate (TpCP), TDCPP and TCPP, along with their respective metabolites, bis(2-chloroethyl) phosphate (BCEP), diphenyl phosphate (DPHP), di-o-cresyl phosphate (DoCP), di-m-cresyl phosphate (DmCP), di-p-cresyl phosphate (DpCP), bis(1,3-dichloro-2-propyl) phosphate (BDCPP) and bis-(1-chloro-2-propyl) phosphate (BCPP). Figure 1 presents the chemical structures of the parent OPFRs and their corresponding metabolites investigated in this study. The investigated urinary metabolites have been described as biotransformation products and/or biomarkers of exposure to their corresponding parent OPFRs [21,22,23,24]. Detailed information on the analytical standards, chemicals, and reagents is provided in Supplementary Information (File S1).

### 2.2. Study Population, Ethics and Urine Collection

All the participants in this study were originally enrolled in a wide-ranging project conducted by the French National Research and Safety Institute for the Prevention of Occupational Accidents and Diseases (INRS) to assess occupational exposure to FRs in WEEE sites in France in 2023. The human biomonitoring part of this project was approved by the “EstII” Ethics Committee under the reference number 2022-A01643-40. To assess the temporal variability of OPFR concentrations, six exposed workers were requested to provide all spot urine samples collected over one workweek from Monday to Saturday morning. All the participants were given information about the study and provided written informed consent. The group included three workers from an electronic board site and three workers handling small household appliances (small HHAs). All WEEE workers were male, with a median age of 49.5 years (range: 37–53). In accordance with the approved ethical protocol, participation was voluntary; consequently, only six workers were included and a low number of samples were missing. A total of 111 urine samples were collected during the workweek and stored at −20 °C until analysis.

### 2.3. Sample Preparation and Analysis

A multi-target urinary method was developed and validated for OPFRs and their metabolites, except for BCEP, which required a specific method due to its high polarity [25]. Enzymatic hydrolysis optimization and sample extractions are described in Supplementary Information (File S1.pdf) [22,26,27,28,29,30]. Particular attention was paid to controlling contamination along the analytical process [31]. Note that, for both methods and to compensate for the matrix effects, labelled standards and matrix matched calibration were used, by supplementing a pool of urine at eight different concentration levels, ranging from 0.1 to 125 µg/L, depending on the compound; and by then applying the same extraction procedure (File S2-Table S1). Urine extracts were analyzed by liquid chromatography-tandem mass spectrometry (LC-MS/MS) using a Waters Acquity UPLC H-Class system coupled with a Waters Xevo TQ-S micro tandem mass spectrometer (Waters, Milford, MA, USA), equipped with an electrospray ion source. OPFR parent compounds and their metabolites were separated using a BEH C18 reversed-phase column and analyzed in multiple reaction monitoring (MRM) mode using positive and negative electrospray ionization. Detailed chromatographic conditions, mass spectrometry parameters, MRM transitions, quality control and method validation parameters, established according to recommended analytical validation procedures [32,33], are provided in Supplementary Information (Files S1 and S2-Table S2). Due to insufficient chromatographic separation of DmCP and DpCP, these two metabolites were quantified and reported as their combined concentration (DmCP + DpCP).

### 2.4. Statistical Analysis

All statistical analyses were performed using the Stata (v18.0) software and the significance level was set at 5%. The urinary OPFR metabolite concentrations used for all statistical analyses were normalized to specific gravity (SG), using the sample mean SG to account for urinary dilution, as described elsewhere [12].

#### 2.4.1. Descriptive Statistical Analysis

Descriptive statistical analysis was performed on the OPFR concentrations in the collected urine samples (reporting the median, maximum and frequencies (%) of detection).

#### 2.4.2. OPFR Urinary Elimination Profiles

- Representation of OPFR urinary elimination profiles over time. The urinary elimination profile of OPFRs were assessed by graphically describing urinary concentration measurements over the working period.
- Assessment of OPFR intra- and inter-day variability using Intra-Class Correlation coefficient (ICC). A log transformation was applied to urinary OPFR concentrations to normalize the distributions. Assessment of OPFR variabilities was based on the ICC coefficient estimations. To this end, the weekly mean OPFR level of each worker (on the log scale) was subtracted from their individual spot urinary concentrations (also on the log scale) for the between-subject variability to be corrected [34]. A mixed linear regression model was then applied to the log-transformed OPFR concentrations including a random effect “day” to account for variability between day and combined with an interval regression (Tobit-type) model to account for censored measurements below the LOD or between the LOD and the LOQ [35]. No substitution was applied to values below the LOQ for statistical analyses. For graphical representation only, concentrations below the LOQ were replaced by LOQ/2.

ICC was defined as the ratio of the between-day variance to the total variance (the sum of the inter-day and intra-day variance). An ICC < 0.4 indicates poor reproducibility of urinary OPFRs within the day while 0.4 ≤ ICC < 0.75 indicates fair to good reproducibility and, ICC ≥ 0.75 indicates good reproducibility [14,34].

- Assessment of the most representative spot urine sample for accurate evaluation of occupational exposure. Occupational human biomonitoring (HBM) studies usually consider pre-shift urine as control urine. The urine samples are generally classified as FMV (first morning void), pre-shift urine (collected between 2 h before and 2 h after the start of the shift), post-shift urine (collected 2 h before or 2 h after the end of the shift), nightfall urine (collected before sleep) and finally, all other types (collected at other times during the working day). A statistical analysis was carried out, for each metabolite, to evaluate the difference between each specific time-sampled urine specimen (post-shift, nightfall, FMV, others) and the pre-shift urines. For each OPFR, a mixed linear regression model, combining a “sampling time” fixed effect and a “subject” random effect, was performed on the log-transformed OPFR concentrations and was associated with an interval regression model. When a significant fixed effect was observed, Bonferroni-corrected post-hoc tests were performed to compare pre-shift and other time-sampled urines.

The same statistical analysis was repeated for each metabolite by means of a “refined definition” of sample collection time, which categorized urine samples according to their collection time relative to the end of the worker’s shift. In this classification, hour 0 corresponds to the end of the work shift. The refined classification of urine is summarized in Table 1, where each category is described along with its corresponding time window and potential equivalent in general human biomonitoring.

Finally, the same statistical analysis was applied, with a “Working day” fixed effect followed by a post-hoc comparison of metabolite concentrations on the first working day with those on subsequent days to identify metabolites showing an accumulation profile over the workweek.

## 3. Results and Discussion

### 3.1. OPFR Urinary Levels of the Study Population

None of the parent OPFR compounds were detected in any of the urine samples. A summary of unadjusted and SG-adjusted urinary concentrations (ng/mL) of OPFR metabolites, including detection frequencies (DF, %), medians, and maximum values, is presented in Table 2. Unadjusted concentrations were used only for descriptive comparison with previously published studies reporting concentrations on the same basis. Creatinine-adjusted concentrations (µg/g Cr) are provided in Supplementary Information File S2-Table S3 to facilitate comparison with studies using creatinine-based correction for urinary dilution. Among all urine samples (*n* = 111), DPHP, DmCP + DpCP, BDCPP, and BCEP were frequently detected, with detection frequencies of 100%, 99%, 98%, and 80%, respectively. In contrast, BCPP and DoCP showed markedly lower detection frequencies (29% and 1%, respectively), indicating limited occurrence among the study workers, therefore, these metabolites were excluded from further analysis. Median concentrations were 2.55, 0.38 and 0.35 ng/mL for DPHP, DmCP + DpCP and BDCPP, respectively, whereas BCEP concentrations were generally below the limit of quantification (LOQ). Maximum concentrations reached 9.52 ng/mL for DPHP, 2.11 ng/mL for DmCP + DpCP, 1.31 ng/mL for BDCPP and 1.61 ng/mL for BCEP.

The unadjusted urinary concentrations of OPFR metabolites measured in WEEE workers were compared with the values reported in the literature for both general and occupationally exposed populations (File S2, Table S4) [12,19,21,30,36,37,38,39,40,41,42,43,44,45,46,47,48,49,50,51,52,53,54,55]. Overall, the median DPHP concentration in our study (2.55 ng/mL) was perceptibly higher than the levels reported in general population studies from the USA and Europe [12,21,30,40,42,47,53,54,55], although higher concentrations have been reported in other populations [52]. The present BDCPP concentrations among WEEE workers (median: 0.35 ng/mL) were comparable to those reported in the general European population [12,47,53,55] but lower than those observed in some occupational settings [19,30,37,39,40,47,48,50] (File S2, Table S4). Upon comparison with other occupational groups, our results suggest that WEEE workers have moderate exposure levels, lower than those observed in highly exposed populations such as firefighters and spray polyurethane foam workers [30,37]. DmCP + DpCP concentrations were low albeit detectable (median: 0.38 ng/mL), unlike many general population studies in which these metabolites were frequently below the LOD [30,54,55]. Similarly, several occupational studies either failed to detect di-cresyl phosphate (DCP) metabolites or reported concentrations that were largely below detection or quantification limits [39,43], although low levels have been reported in some e-waste and industrial worker populations [42,49].

Focusing specifically on the e-waste sector, the present DPHP concentrations were 1.5 times higher than those reported in e-waste recycling workers in Canada [36] and 4.5 to 23-fold higher than those reported in dismantling or recycling workers in China [41] while BDCPP levels were broadly comparable. Differences across studies may reflect variations in waste composition, processing practices, or regulatory contexts and study populations. However, these comparisons should be considered descriptive and interpreted with caution rather than as representative estimates of occupational exposure levels, especially by reason of the low number of workers included in the present pilot study.

### 3.2. OPFR Urinary Elimination Profiles

#### 3.2.1. OPFR Urinary Elimination Profiles over Time

**Figure 2:**
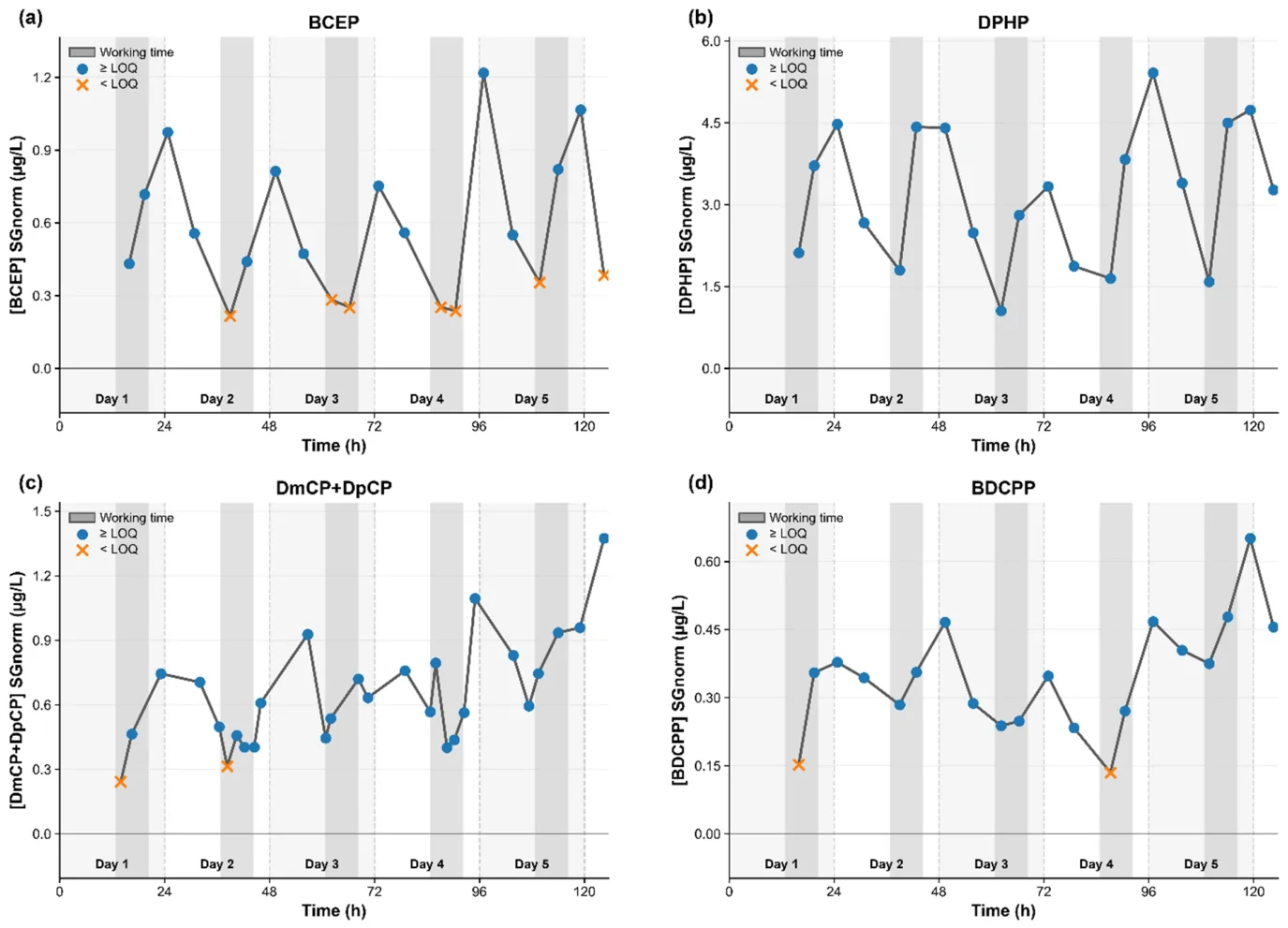
Urinary elimination profiles of OPFRs over one workweek for an exposed worker. (**a**) BCEP, (**b**) DPHP, (**c**) DmCP + DpCP and (**d**) BDCPP.

The first assessment of urinary temporal variability was achieved by plotting the urinary concentration of each quantified metabolite for each individual over the working week, together with the corresponding exposure periods. Two major elimination profiles were observed. The first profile, which was common to the six exposed workers, was observed for DPHP and BCEP. Higher urinary concentrations were detected after the end of the work shift, and these concentrations decreased to baseline levels by the following pre-shift working day (Figure 2a,b). The second elimination profile was characterized by an increase in DmCP + DpCP urinary concentrations during the working week (Figure 2c). The elimination profile of BDCPP was less well-defined, showing an apparent accumulation over the week with fluctuations in urinary concentration. Consequently, the overall pattern remained unclear, making it difficult to draw firm conclusions.

These urinary elimination profiles highlight the necessity to select more representative exposure sampling times that are specific to each metabolite. For instance, DmCP + DpCP shows a gradual accumulation pattern over the week, while DPHP and BCEP exhibit acute post-shift increases followed by rapid declines in urinary concentrations. A more comprehensive statistical analysis was therefore required to confirm these findings based on individual-level data.

#### 3.2.2. Temporal Variability of Urinary Excretion of OPFRs over the Working Week

The intra- and inter-day variabilities of each OPFR metabolite were assessed using the day-ICCs of the exposed workers based on the log-transformed OPFR concentrations, which were adjusted for specific gravity. Table S5 presents the ICC obtained for each metabolite with a detection frequency greater than 80%.

Poor intra-day reproducibility was observed for BCEP, DPHP and BDCPP (ICC < 0.2), with intra-day variability being much higher than inter day-variability. When comparing these findings with previously reported ICCs, differences in study populations, exposure settings, and sampling designs should be considered. Previous studies were conducted primarily in general populations, whereas our work-shift-based sampling targeted occupationally exposed workers. Nevertheless, these studies provide useful context for interpreting our findings. In a repeated-sampling study over seven consecutive days, ICCs for OPFR metabolites ranged from 0.042 to 0.349, with intra-day variability contributing substantially to total variability [14]. Similarly, Bastiaensen et al. (2021), who collected spot urine samples from 10 adults over five consecutive days, reported lower temporal reproducibility for DPHP (ICC = 0.303; Table S6) and found that between-day variability was generally minor compared with the within-day variability [12]. In contrast, higher reproducibility was reported for BDCPP in that study and for BDCPP and BCEP by Wang et al. (2019), although the latter assessed inter-day rather than intra-day variability [12,56].

Comparison with available toxicokinetic data should also be interpreted cautiously, as human half-life estimates for OPFRs remain uncertain and vary across studies (Table S6) [12,25,27,28,29,30,40,52,54,56,57,58,59,60,61,62,63,64]. For instance, Wang et al. (2020) estimated biological half-lives of TPHP, TCEP, and TDCPP to be 9.58, 17.4, and 53.8 days, respectively [10]. However, substantial differences in these estimates have been reported. Bui et al. (2017) estimated the half-life of TPHP at less than 2 h [57], compared with 9.58 days by Wang et al. (2020) [10]. Based on currently available published data, a direct relationship between parent-compound half-life and urinary metabolite ICC cannot be established.

#### 3.2.3. Assessment of the Most Representative Spot Urine Sample for Accurate Evaluation of Occupational Exposure

**Figure 3:**
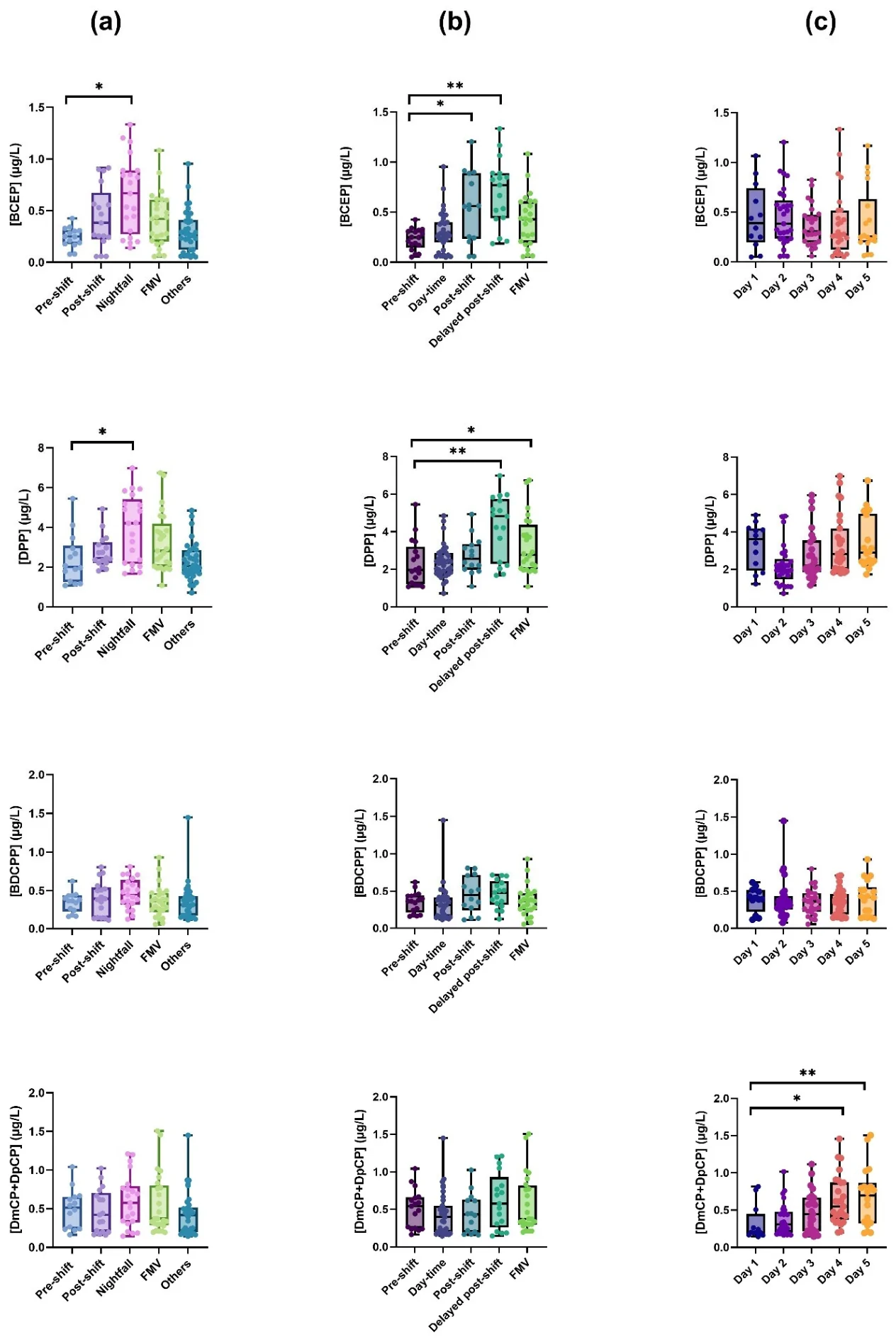
Box plots of SG-corrected concentrations of the four metabolites in spot urine samples from exposed workers according to sampling time criteria. (**a**) Conventional HBM definition, (**b**) Refined definition based on time elapsed from the end of the shift. (**c**) Groups based on weekly working days. Boxes show median, interquartile range and potential outliers for each group. Asterisks (*) indicate statistically significant differences from first group (Bonferroni-corrected post-hoc test after linear mixed-effects interval regression on log-transformed OPFR concentrations with “sampling time” fixed effect and subject random effect) (*: *p*-value < 0.05, **: *p*-value < 0.01), FMV: First Morning Void.

Comparison was performed between the classical spot urine sampling times usually defined in occupational HBM studies (see Section 2.4.2). Figure 3a shows the boxplots of concentrations of each metabolite according to these predefined collection times. Based on this classification, a single significant difference was observed: nightfall urine showed significantly higher concentrations than pre-shift urine for both BCEP and DPHP. These results suggest that nightfall urine could be a suitable spot urine sample for evaluating occupational exposure for these two OPFR metabolites.

However, the timing of a given spot urine category (e.g., “nightfall urine”) can vary according to individuals, work shifts (morning, day or night) and studies. To account for this variability, a second classification was applied based on the time elapse between the end of each individual’s shift and urine collection. This approach allowed more precise evaluation of sampling windows reflecting occupational exposure. When “pre-shift” samples were compared with the reclassified time points (Table 1), the most significant differences for both BCEP and DPHP were observed in delayed post-shift specimens (Figure 3b). Our DPHP results are consistent with a previous study of collegiate gymnasts which reported higher urinary DPHP concentrations 30 min after practice than pre-practice levels. In our study, urine collected from 2 to 5 h after the end of the shift appeared to best reflect occupational exposure, supporting the relevance of post-activity sampling for capturing recent DPHP exposure [19]. For BCEP, however, the observed patterns should be interpreted with caution. The relatively lower frequency quantification, combined with the limited sample size, increases uncertainty regarding differences between sampling times, making conclusions about the most representative spot urine for BCEP less robust than those for DPHP.

A final approach was used to process the data and thus identify the most representative spot urine sample for accurately assessing occupational exposure. The latter consisted in comparing working days, which appeared to be more suitable for metabolites showing an accumulation profile. The same statistical analysis was therefore performed, comparing spot urine samples collected on the first working day with those collected on subsequent days (Figure 3c). One significant difference was observed for DmCP + DpCP after four working days, with the greatest difference measured on the last day of the workweek. Based on these results, these metabolites appear to exhibit a longer elimination profile with accumulation throughout the working week. Consequently, the spot urine time which was the most representative of occupational exposure to this OPFR appears to be the end of the working week.

Regarding BDCPP, no significant differences were observed between any of the evaluated sampling times (Figure 3). Several hypotheses may account for this result. Firstly, occupational exposure to the parent compound (TDCPP) may be limited, resulting in concentrations that are too low to produce a measurable difference between pre-shift urine and any other spot urine samples. Secondly, BDCPP levels may reflect accumulation due to the longer half-life of TDCPP (54 days), as previously suggested, implying that the timing of spot urine collection may be less critical for this metabolite [10,50]. Finally, the ubiquitous presence of OPFRs, particularly TDCPP in indoor environments, may result in substantial background exposure that masks potential work-related differences. As reported by Gravel et al. (2025), no significant association was found between TDCPP concentrations in the air and urinary BDCPP levels in electronic waste recycling workers [36].

These results underscore the importance of identifying the most representative urine sampling for occupational biomonitoring to minimize misclassification. Previous studies have similarly emphasized the importance of appropriate sampling strategies for reliable exposure assessment in occupational settings [12,65]. In this study, distinct elimination profiles were therefore identified for the targeted metabolites. Both BCEP and DPHP exhibited pronounced short-term variations, with both compounds predominantly detected within 2 to 5 h after the work-shift. These findings indicate that the timing of urine collection may be particularly important for capturing occupational exposure to these compounds. In contrast, DmCP + DpCP progressively increased over the workweek, demonstrating greater inter-day than intra-day variability. Consequently, samples collected only at the beginning of the week may show no significant difference in DmCP + DpCP concentrations between pre-shift and post-shift, whereas comparisons across the workweek, particularly on days 4 and 5, reveal a progressive increase. These contrasting profiles highlight that the most informative sampling time may differ among OPFR metabolites.

These compound-specific temporal profiles may be due to several factors, including physicochemical properties and chemical structure, as well as differences in exposure timing and frequency, exposure route, absorption, metabolism, distribution, and urinary excretion. The difference in lipophilicity was observed among the compounds (see Table S6): BCEP and DPHP are relatively less lipophilic showing pronounced short-term fluctuations, whereas DmCP and DpCP are more lipophilic progressively increasing over the workweek. Their higher lipophilicity (log Kow~3.7) may contribute to slower systemic elimination and longer persistence, potentially accounting for their more gradual accumulation compared with BCEP and DPHP. Differences in exposure routes may also contribute to these patterns. In particular, dermal uptake may be relevant for OPFRs, and experimental studies have demonstrated compound-specific variation in skin penetration and retention. Greater lipophilicity may favor skin retention and delayed absorption [66]. In occupational setting with repeated exposure, these factors could contribute to the distinct urinary profiles observed among metabolites. However, the present biomonitoring data cannot determine their relative contribution. These findings highlight the importance of considering compound-specific temporal variability when selecting urine sampling times for occupational biomonitoring.

Overall, these results highlight the need to characterize metabolite elimination profiles to ensure reliable spot urine sample collections in occupational settings where 24-h urine collection is generally not feasible. Such identification is, however, particularly challenging given the limited data currently available on the elimination profiles and half-lives of relevant OPFR metabolites. The methodology used in this study addresses this gap, providing a practical framework for selecting the most appropriate sampling strategy. In this refined approach, pre-shift samples were collected 7 to 9 h before the end of the shift, while delayed post-shift samples were collected 2 to 5 h after the end of the shift, rather than at the conventional start- and end-shift time points. For DPHP and BCEP, this adjusted timing resulted in more pronounced differences in urinary concentration, thereby increasing the sensitivity of exposure assessment. Thus, metabolites such as DPHP and BCEP, which showed low short-term reliability, require particular attention because reliance on a single spot urine sample may substantially affect exposure assessments estimates. Conversely, the gradual increase in DmCP + DpCP levels suggests that samples collected at the end of the workweek may better reflect occupational exposure to TmCP and TpCP than samples collected only at the beginning of the week.

Previous research has examined the temporal variability of OPFR metabolites in the general population [12,19]. However, comparable investigations in occupational settings remain limited. To our knowledge, this study is among the first ones to investigate the temporal variability of urinary OPFR metabolites using multiple urine samples collected in an occupational exposure context, specifically in a real-world workplace environment. Our findings demonstrate that the most informative urine sampling time may differ among metabolites, emphasizing the importance of characterizing compound-specific temporal profiles to minimize exposure misclassification. Given the limited kinetic information currently available for many OPFRs, the approach applied here provides a practical framework for identifying appropriate metabolite-specific sampling times for occupational biomonitoring.

Strengths and limitations: This study has several limitations that should be acknowledged. Firstly, the number of metabolites analyzed was limited for certain OPFRs, hydroxylated metabolites not being included due to the availability of analytical standards. The development of target analytical methods for these metabolites will be essential to achieving a more comprehensive characterization of OPFR exposure. Secondly, the sample size for the temporal variability assessment was relatively small (*n* = 6), reflecting the practical challenges of recruitment in occupational settings, particularly when repeated urine sampling is required. This limited sample size reduces statistical power and may not capture inter-individual variability related to factors such as age, metabolism, or specific occupational tasks. Therefore, these findings should be considered preliminary and interpreted within the context of this methodological pilot study. In addition, all exposed participants were men, which may limit the extrapolation of the findings to female workers. Despite these limitations, this study enabled the identification of an optimal and practical sampling strategy, demonstrating that spot urine samples collected between 2 and 5 h after the end of the shift, particularly at the end of the workweek, may provide a more representative measure of occupational exposure for the selected OPFR metabolites. Furthermore, the concentrations of DPHP and DmCP+ DpCP were higher than those reported in non-occupational populations, and exceeded levels previously observed in occupational e-waste settings. These findings emphasize the need for a broader investigation within the WEEE sector and a more comprehensive assessment of occupational exposure to OPFRs. Accordingly, the validated sampling strategy was implemented in the following phase of the project, which involved approximately 60 workers and 30 controls from six WEEE companies. This approach, based on optimized spot urine sampling, is more acceptable to participants than 24-h urine collection or repeated spot urine sampling strategies. An expanded study such as this one would strengthen our understanding of OPFR-related health risks and contribute to improved worker safety and regulatory practices.

## 4. Conclusions

This study highlights the critical importance of understanding the elimination profile of OPFRs in e-waste workers to optimize biomonitoring strategies. Variability in OPFR metabolite excretion observed along the workday reveals the limitations of relying on single spot urine samples, as this may lead to underestimation of exposure levels. Although 24-h urine collection or repeated sampling can reduce this variability, such approaches are often impractical. Therefore, identifying the most representative spot urine sample time is essential for balancing accurate exposure assessment with feasibility in occupational settings.

By investigating the urinary excretion profiles of five OPFR metabolites, our conclusions provide practical guidance on optimal urine collection timing. Specifically, we recommend prioritizing sample collection later in the workweek for DmCP + DpCP, as this study is among the first to examine their temporal variability in a real-world occupational setting and to report an accumulation pattern, suggesting a potentially longer elimination profile for this metabolite. In contrast, 2 to 5 h delayed post-shift sampling after the end of the workday appears more suitable for DPHP and BCEP. These targeted sampling strategies combining end-of-workweek collection with 2 to 5 h post shift delay when appropriate, may improve the accuracy of exposure assessments while reducing the need for excessive sample collection, thereby making biomonitoring studies more efficient and cost-effective.

Furthermore, our findings underscore the need for additional elimination profile studies on other OPFRs. Such studies will improve the specificity of exposure assessments, refine sampling strategies, and expand the applicability of the present conclusions. While this study provides valuable insights, its findings are limited by a focus on a restricted set of OPFRs, which prevents generalization. Future research should encompass a wider array of flame retardants and address the challenge of identifying specifically exposed individuals, as information on exposure is often lacking.

## Figures and Tables

**Table 1:** Refined classification of urine samples based on collection time relative to end of work shift. Each category was defined by a specific time window and described along with its potential equivalent in HBM studies.

Refined Classification	Time of Collection (Relative to Shift End)	Description
Pre-shift	−9 to −7 h	Collected well before the work shift; considered pre-exposure
Day-time	−7 to −1 h	Collected during the workday, prior to shift end
Post-shift	~−1 to +1 h	Collected immediately before or after the end of the shift
Delayed post-shift	+2 to +5 h	Collected a few hours after the end of the shift
FMV	>+10 h	First morning void on the following day

**Table 2:** Detection frequency (DF in %), median, and maximum of unadjusted urinary concentrations and normalized to SG (ng/mL) of organophosphate ester metabolites across participants.

	FR Metabolites	DPHP	DmCP + DpCP	BDCPP	BCEP	BCPP	DoCP
Unadjusted-urine							
Total number of samples (*n* = 111)	DF (%)	100	99	98	80	29	1
	Median (ng/mL)	2.55	0.38	0.35	<LOQ	<LOD	<LOD
	Max (ng/mL)	9.52	2.11	1.31	1.61	0.93	0.91
Electronic boards samples (*n* = 52)	DF (%)	100	98	96	71	6	0
	Median (ng/mL)	2.40	0.41	0.28	<LOQ	<LOD	<LOD
	Max (ng/mL)	6.90	1.60	0.89	1.13	0.93	<LOD
Small HHA samples (*n* = 59)	DF (%)	100	100	100	88	49	2
	Median (ng/mL)	2.98	0.34	0.38	<LOQ	<LOD	<LOD
	Max (ng/mL)	9.52	2.11	1.31	1.61	0.72	0.91
SG-adjusted urine							
Total number of samples (*n* = 111)	DF (%)	100	99	98	80	29	1
	Median (ng/mL)	2.46	0.44	0.36	<LOQ	<LOD	<LOD
	Max (ng/mL)	6.99	1.51	1.45	1.33	1.50	0.87
Electronic boards samples (*n* = 52)	DF (%)	100	98	96	71	6	0
	Median (ng/mL)	2.09	0.47	0.32	<LOQ	<LOD	<LOD
	Max (ng/mL)	6.62	1.46	1.45	1.08	1.50	<LOD
Small HHA samples (*n* = 59)	DF (%)	100	100	100	88	49	2
	Median (ng/mL)	2.97	0.34	0.41	<LOQ	<LOD	<LOD
	Max (ng/mL)	6.99	1.51	0.93	1.33	1.17	0.87

*n*: number of samples; <LOD: below limit of detection; <LOQ: below limit of quantification; SG: Specific Gravity; Urine concentration normalized based on its specific gravity; HHA: household appliances.

## Data Availability

The data supporting this study are available from the corresponding author upon request.

## Supplementary Materials

The following supporting information can be downloaded at: https://www.mdpi.com/article/10.3390/toxics14090778/s1, File S1.pdf: Additional information on sample preparation and analysis, including chemicals, sample preparation, enzymatic hydrolysis test, urine sample extraction, LC-MS/MS analysis, and quality control/method validation; Figure S1: Linear model prediction of DPHP concentration using different enzymes (H1, HP-2, IX-A) at different doses (1000, 5000, 10,000, and 20,000 U) and incubation times (2, 4, 16, and 20 h). The model applied was a linear regression model with a third-order interaction. File S2.xlsx. Six tables summarizing sample preparation and method validation, additional biomonitoring results, comparisons with the literature, and physicochemical and toxicokinetic information for the investigated compounds. Table S1: Multi-concentration stock solution mixture in ACN and calibration range; Table S2: Instrument parameters and summary of method validation; Table S3: Detection rates (%), median, and maximum urinary concentrations of organophosphate ester metabolites normalized to creatinine (µg/g Cr) across participants; Table S4: Urinary organophosphate ester metabolite concentrations (ng/mL) reported in the literature for non-occupationally and occupationally exposed adults; Table S5: Day-ICC and variance distribution of log-transformed specific-gravity-adjusted OPFR metabolite concentrations in samples from exposed workers; Table S6: Overview of physicochemical properties, conjugation, urinary excretion, and toxicokinetic parameters of the investigated compounds.

## Abbreviations

BCEP	Bis(2-chloroethyl) phosphate
BCPP	Bis-(1-chloro-2-propyl) phosphate
BDCPP	Bis(1,3-dichloro-2-propyl) phosphate
BFRs	Brominated Flame Retardants
Cr	Creatinine
DCP	Di-cresyl phosphate
DF	Detection frequencies
DmCP	Di-m-cresyl phosphate
DoCP	Di-o-cresyl phosphate
DpCP	Di-p-cresyl phosphate
DPHP	Diphenyl phosphate
EEE	Electrical and Electronic Equipment
EU	European
FMV	First Morning Void
FR	Flame retardants
HBM	Human biomonitoring
HHAs	Household appliances
ICC	Intraclass correlation coefficient
INRS	French National Research and Safety Institute for the Prevention of Occupational Accidents and Diseases
LC-MS/MS	Liquid chromatography–tandem mass spectrometry.
LOD	Limit of detection
LOQ	Limit of quantification
OPEs	Organophosphate esters
OPFR	organophosphate flame retardants
PBPK	Physiologically Based Pharmacokinetic
SG	Specific gravity
TCEP	Tris(2-chloroethyl) phosphate
TCPP	Tris(1-chloro-2-propyl) phosphate
TDCPP	Tris(1,3-dichloro-2-propyl) phosphate
TmCP	Tri-m-cresyl phosphate
ToCP	Tri-o-cresyl phosphate
TpCP	Tri-p-cresyl phosphate
TPHP	Triphenyl phosphate
WEEE	Waste Electrical and Electronic Equipment
