# Supplementary material for: Temporal Variability of Urinary Organophosphate Flame Retardant Metabolites Among E-Waste Workers in France: A Pilot Study to Inform Occupational Biomonitoring Sampling Design
Source: Toxics. 2026 Aug 31;14(9):778. doi: 10.3390/toxics14090778 (PMC13611014; doi:10.3390/toxics14090778)
Supplement: Supplementary file 1 [file toxics-14-00778-s001.zip › File S1.pdf]

Supplementary data

Temporal Variability of Urinary Organophosphate Flame Retardant Metabolites Among E-Waste Workers in France: A Pilot Study to Inform Occupational Biomonitoring Sampling Design

Baninia Habchi [1,*,†], Guillaume Antoine [1,†], Aurélie Martin Remy [1], François Zimmermann [2], Marie-Thérèse Lecler [2], Williams Esteve [3], Claude Emond [4,5], Nathalie Grova [1] and Sophie Ndaw [1]

[1] Department of Toxicology and Biomonitoring, INRS (French National Research and Safety Institute for the Prevention of Occupational Accidents and Diseases), CS 60027, 54519 Vandoeuvre-lès-Nancy Cedex, France; guillaume.antoine@inrs.fr (G.A.); aurelie.remy@inrs.fr (A.M.R.); nathalie.grova@inrs.fr (N.G.); sophie.ndaw@inrs.fr (S.N.)

[2] Department of Process Engineering, INRS (French National Research and Safety Institute for the Prevention of Occupational Accidents and Diseases), CS 60027, 54519 Vandoeuvre-lès-Nancy Cedex, France; francois.zimmermann@inrs.fr (F.Z.); marie-therese.lecler@inrs.fr (M.-T.L.)

[3] Department of Pollutants Metrology, INRS (French National Research and Safety Institute for the Prevention of Occupational Accidents and Diseases), CS 60027, 54519 Vandoeuvre-lès-Nancy Cedex, France; williams.esteve@inrs.fr

[4] Department of Occupational and Environmental Health, School of Public Health, Université de Montréal, Montréal, QC H3N 1X9, Canada; claude.emond@umontreal.ca

[5] PKSH Inc., Crabtree, QC J0K 1B0, Canada

[*] Correspondence: baninia.habchi@inrs.fr
[†] These authors contributed equally to this work.

Table of contents

1. Materials and methods 3
1.1. Chemicals.. 3
1.2. Sample preparation 3
1.2.1. Enzymatic hydrolysis test 3
1.2.2. Urine sample extraction 5
1.3. LC-MS/MS analysis 6
1.4. Quality control/method validation.. 7
References 9

1. Materials and methods

1.1. Chemicals

All OPFR parent standards, including tris(2-chloroethyl) phosphate (TCEP), triphenyl phosphate (TPHP), tri-o-cresyl phosphate (ToCP), Tri-m-cresyl phosphate (TmCP), tri-p-cresyl phosphate (TpCP), tris(1,3-dichloro-2-propyl) phosphate (TDCPP) and tris(1-chloro-2-propyl) phosphate (TCPP), along with their respective metabolites, bis(2-chloroethyl) phosphate (BCEP), diphenyl phosphate (DPHP), di-o-cresyl phosphate (DoCP), di-m-cresyl phosphate (DmCP), di-p-cresyl phosphate (DpCP), bis(1,3-dichloro-2-propyl) phosphate (BDCPP) and bis-(1-chloro-2-propyl) phosphate (BCPP), were purchased from LGC Standards (Molsheim, France) with purity exceeding 95 %. Internal standards, including TCEP-d12, BCEP-d8, TPHP-d15, DPHP-d10, TpCP-d21, DpCP-d14, TDCPP-d15, BDCPP-d10, TCPP-d18 and BCPP-d12, were also obtained from LGC Standards (Molsheim, France) with purity exceeding 95 %.

Glacial acetic acid, sodium acetate trihydrate, acetonitrile (ACN), methanol (MeOH), isopropanol, ultrapure water, ammonium hydroxide and formic acid were sourced from VWR (Strasbourg, France). Acetic acid, used as a mobile phase additive, was procured from Fisher Scientific (Illkirch, France). Solvents and additives used for mobile-phase preparation were of LC-MS grade whereas all other reagents were at least analytical grade. β-glucuronidase from Helix pomatia type HP-2 (>100,000 U/mL) was purchased from Sigma Aldrich (St-Louis, USA). The SPE extraction cartridges (Oasis WAX 60 mg, 3 cc) for urine samples were obtained from Waters (Milford, MA, USA).

1.2. Sample preparation

1.2.1. Enzymatic hydrolysis test

According to the literature, metabolites of flame retardants (FRs) are present in urine in both conjugated and unconjugated forms (Petropoulou et al., 2016). We therefore tested several deconjugation protocols on pooled post-shift urine samples to determine whether an additional hydrolysis step was necessary. The pool sample was processed without and with enzymatic hydrolysis, testing different enzyme sources, concentrations and deconjugation times. The enzymes tested were H1 type β-glucuronidase from Helix pomatia, HP2 type β-glucuronidase from Helix pomatia, and IX-A type β-glucuronidase from E. coli. Enzymatic hydrolysis was performed at concentrations ranging from 1 U/µL to 20 U/µL, as recommended by the supplier, specifically at 1, 5, 10, and 20 U/µL. The samples were incubated at 37°C for various periods of time: 0.5, 2, 4, 16, and 20 hours for each enzyme type.

Statistical analyses were performed to evaluate the effects of enzymatic hydrolysis under different conditions (3 enzymes, 4 incubation times and 4 doses). Firstly, a one-way ANOVA was performed on

the urinary concentration of each metabolite to determine whether the control group (without enzymatic hydrolysis) differed from the other groups (each treated with a specific enzyme at a specific dose and time). This was followed by a multiple comparison test (Bonferroni correction) to compare the control group and the enzymatic hydrolysis groups. Secondly, a linear regression model was applied to the urinary concentration of the molecule for metabolites with a control group that differed from the enzymatic hydrolysis groups. This model included the three main effects of enzyme, dose and time, as well as their second- and third-order interactions, based on the concentrations of each molecule. Where significant interactions were identified, the simple effect of one factor was examined in relation to the levels of the other two factors.

Four urine metabolites were detected in the pooled urine (BCEP, DPHP, BDCPP and the meta and para isomers of DCP which were quantified under the same chromatographic peak (DmCP + DpCP)). DoCP and BCPP were not detected under any conditions. The results showed that hydrolysis did not affect the concentrations of BCEP, BDCPP and DmCP + DpCP, which is consistent with previous studies on BDCPP and BCEP (Chen et al., 2022; Cooper et al., 2011; Jayatilaka et al., 2019; Jayatilaka et al., 2017; Van den Eede et al., 2013a). By contrast, DPHP appeared to be present in a conjugated form, as its concentration increased significantly when treated with an enzyme. Of the three enzymes tested, the H1 enzyme produced the lowest intensity of this metabolite at any time and dose, compared to HP-2 and IX-A. Regarding IX-A and HP2, we observed similar results, with maximum intensity between 16 and 20 h (**Figure S1**). Statistical analysis also indicated consistent intensity across different doses, particularly for the HP-2 enzyme at 16 h. Consequently, these results confirm that DPHP is present in a conjugated form in urine. For optimal quantification and optimal conditions providing the highest intensity at the lowest cost, the HP 2 enzyme, at a dose of 1000 U, and with an incubation time of 16 h, was selected.

The increase in DPHP intensity in enzyme-treated pooled urine samples, compared with untreated samples, aligns with prior studies evaluating optimal conditions for enzymatic treatment (Jayatilaka et al., 2017). In addition, our results highlight the importance of selecting the appropriate enzyme type and optimizing conditions. These findings emphasize the need to evaluate different enzymes and treatment conditions, as these can significantly impact hydrolysis outcomes.

However, the absence of BCPP and DoCP in the tested urine pool restricts our ability to assess the impact of enzymatic hydrolysis on these particular metabolites. Nevertheless, both metabolites are measured using the multi-targeted method, meaning that applying enzymatic hydrolysis should not affect their detection. Furthermore, according to previous studies, no difference has been observed between the enzyme-treated and untreated urine samples for BCPP (Chen et al., 2022; Jayatilaka et

al., 2019; Jayatilaka et al., 2017; Van den Eede et al., 2013a). It would be useful to conduct similar tests in future biomonitoring studies, particularly in other e-waste recycling facilities where exposure to these flame retardants could be more significant.

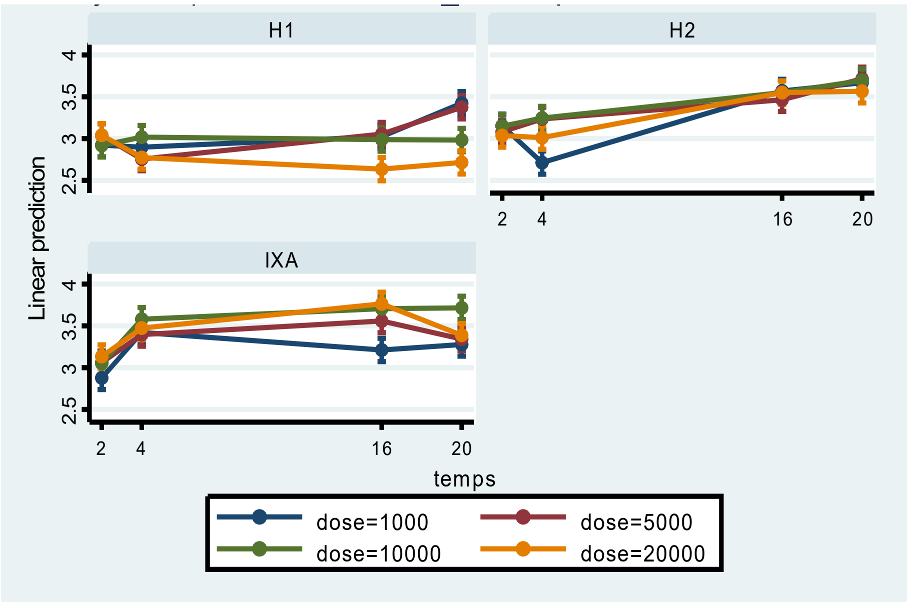

Figure S1: Linear model prediction of concentration of DPHP using different enzymes (H1, HP-2, IX-A) at different doses (1000, 5000, 10000, 20000 U) and incubation times (2, 4, 16, 20 h). Model applied was a regression linear model with third-order interaction.

1.2.2. Urine sample extraction

A multi-target urinary method was developed and validated for OPFRs and their metabolites, except BCEP, which required a specific method due to its high polarity (Van den Eede et al., 2013b). Given the ubiquitous nature of OPFRs, particular attention was paid to controlling contamination along the analytical process. To this end, each material used in the described protocols was tested and validated to minimize contamination as far as possible. Furthermore, all reusable equipment was cleaned following a washing procedure specifically designed for contamination management in mass spectrometry (Waters Corporation, 2021).

Multi-targeted urinary method: the method developed was based on that of Van den Eede et al (Van den Eede et al., 2013b). A multi-concentration internal standard mixture was prepared in ACN containing DPHP-d10, DpCP-d14, BDCPP-d10, and TCPP-d18 at 0.5 mg/L; TPHP-d15 and TDCPP-d15 at 2.5 mg/L; and TCEPd12, BCEP-d8, and BCPP-d12 at 5 mg/L. A 1.2 mL of urine sample was placed in a 4 mL glass vial, to which 8 µL were added of the multi-concentration mixture of internal standards, 1182 µL of 0.1 M sodium acetate buffer (pH5) and 10 µL of β-glucuronidase from Helix pomatia type HP-2 corresponding to 1000 U of glucuronidase and 75 U of sulfatase were added. After 10 s of vortex mixing, the preparation was incubated overnight (16 h) in an oven at 37°C to hydrolyze the conjugated

forms. The compounds were then extracted using Oasis WAX SPE cartridges and a Biotage® Extrahera™ SPE preparation robot (Biotage, Uppsala, Sweden). The cartridges were successively conditioned with 2 mL of MeOH/NH_4OH (ammonium hydroxide) 5% v/v and 3 mL of 1 M sodium acetate buffer pH5. Then, 2 mL of the previously prepared mixture corresponding to 1 mL of urine was loaded onto the cartridge. The cartridge was washed with 2 mL of 1 M sodium acetate buffer pH5/MeOH (70/30 v/v). The compounds were then eluted twice with 2 mL of MeOH/NH_4OH (5% v/v), followed by evaporation to dryness under a nitrogen flow at 1.5 L/min at 40°C. The dry extract obtained was reconstituted with 200 µL of a water/MeOH/ACN mixture (95/2.5/2.5, v/v/v). The extract was finally filtered through a 0.22 µm pore size PTFE syringe filter (Restek, Lisses, France), and collected in a glass vial with an insert for liquid chromatography–tandem mass spectrometry (LC-MS/MS) analysis. To prevent potential contamination of equipment and chemicals, a procedural blank was included in each extraction run. The procedural blank was prepared in the same manner as the samples, with the urine replaced with the same volume of water.

BCEP dedicated method: the BCEP method was based on the multi-targeted method, with some slights adjustments to optimize SPE retention and reduce matrix effects. A sample volume of 1.2 mL of urine was placed in a tube, to which were added 8 µL of BCEP-d8 at 5mg/L in ACN and 1192 µL of 0.1 M sodium acetate buffer pH5. After 10s of vortex mixing, the compounds were extracted using Oasis WAX SPE cartridges and a Biotage® Extrahera™ SPE preparation robot (Biotage, Uppsala, Sweden). The same sample-extraction conditions as those used as in the multi-targeted urinary method were applied, except for the cartridges conditioning (2 mL MeOH/NH_4OH 5 % v/v followed by 3 mL of water containing 2 % formic acid) and the washing steps (2 mL of water containing 2 % formic acid followed by2 mL MeOH containing 2 % formic acid).

For both methods and to compensate for matrix effects, labelled standards and matrix matched calibration were used. A urine pool was supplemented at eight different concentration levels, ranging from 0.1 to 125 µg/L depending on the compound, and then subjected to the same extraction procedure (**File S2-Table S1**).

1.3. LC-MS/MS analysis

The sample extracts were analyzed using LC-MS/MS using a Waters Acquity UPLC H-Class system coupled with a Waters Xevo TQ-S micro tandem mass spectrometer (Waters, Milford, MA, USA), equipped with an electrospray ion source. To prevent system contamination and improve sensitivity, a trapping column (Isolator column 50 x 2.1 mm; Waters, Milford, USA) was placed between the mixer and the injector. OPFR parent compounds and their metabolites were separated using a BEH C18

reversed-phase column (50x2.1mm; 1.7µm; Waters, Milford, USA). The mobile phases consisted of (A) water 0.1 % acetic acid and (B) MeOH/ACN 50/50 containing 0.1 % acetic acid. The gradient started at 5 % of B for 0.2 min, then increased to 50 % at 2.5 min, and to 100 % at min 11 until min 14 to wash the column, and then returned to the initial conditions (95/5 A/B) until min 19 for optimal column re-equilibration before the next injection. The flow rate was 0.4 mL/min and the column temperature was maintained at 70 °C. The injection volume was 5 µL. Mass-spectrometer acquisition was performed in MRM mode, with ionization in positive and negative modes (ESI+ and ESI-), depending on the compounds. The MRM transitions of the compounds are shown in **File S2 - Table S1**. The source parameters were as follows: source temperature 120 °C, desolvation temperature 300 °C, cone gas flow rate 1 L/h, desolvation gas flow rate 1000 L/h, capillary voltage in ES+ mode 1 kV, capillary voltage in ES- mode -0.58 kV.

1.4. Quality control/method validation

Method-validation parameters are reported in Supplementary information (**File S2 - Table S2**). External quality control was ensured through successful participation in OSEQAS inter-laboratory comparison exercises (INSPQ, Québec).

To determine the limit of detection (LOD), blank urine and procedural blank were extracted and analyzed as described for real samples under intra- and inter-day conditions (n=10). Then, LODs were set as three times the standard deviation calculated from the blank urine when no analyte was detectable, otherwise the standard deviation calculated from the procedural blanks was used (Magnusson and Örnemark, 2014).

The limit of quantification (LOQ) was defined as the lowest concentration that could be determined with acceptable repeatability (20 %) and accuracy (80-120 %) (Kruve et al., 2015). To determine the LOQs of OPFR metabolites, two different urine pools were supplemented at different concentration levels (**File S2 - Table S1**) and the entire analytical method, including all steps, was then applied under intra- and inter-day conditions (n=10 for each concentration level). For OPFR metabolites already present in the urine pools, LOQ was set as 10 times the standard deviation measured in procedural blanks, under intra- and inter-day conditions (n=10 for each concentration) (Magnusson and Örnemark, 2014).

Internal quality controls were performed by analyzing procedural blanks and two levels of supplemented urine samples (levels 5 and 7, **File S2 - Table S1**). These quality control samples were regularly injected every fifteen samples, and at the end of the analytical sequence. Procedural blanks

which contained traces of analytes were subtracted from the sample concentrations for TPHP, DPHP, TDCPP and TCPP.

References

Chen, Z.F., Tang, Y.T., Liao, X.L., Jiang, J.R., Qi, Z., Cai, Z., 2022. A QuEChERS-based UPLC-MS/MS method for rapid determination of organophosphate flame retardants and their metabolites in human urine. Sci Total Environ 826, 153989.

Cooper, E.M., Covaci, A., van Nuijs, A.L., Webster, T.F., Stapleton, H.M., 2011. Analysis of the flame retardant metabolites bis(1,3-dichloro-2-propyl) phosphate (BDCPP) and diphenyl phosphate (DPP) in urine using liquid chromatography-tandem mass spectrometry. Anal Bioanal Chem 401, 2123-2132.

Jayatilaka, N.K., Restrepo, P., Davis, Z., Vidal, M., Calafat, A.M., Ospina, M., 2019. Quantification of 16 urinary biomarkers of exposure to flame retardants, plasticizers, and organophosphate insecticides for biomonitoring studies. Chemosphere 235, 481-491.

Jayatilaka, N.K., Restrepo, P., Williams, L., Ospina, M., Valentin-Blasini, L., Calafat, A.M., 2017. Quantification of three chlorinated dialkyl phosphates, diphenyl phosphate, 2,3,4,5-tetrabromobenzoic acid, and four other organophosphates in human urine by solid phase extraction-high performance liquid chromatography-tandem mass spectrometry. Anal Bioanal Chem 409, 1323-1332.

Kruve, A., Rebane, R., Kipper, K., Oldekop, M.L., Evard, H., Herodes, K., Ravio, P., Leito, I., 2015. Tutorial review on validation of liquid chromatography-mass spectrometry methods: part I. Anal Chim Acta 870, 29-44.

Magnusson, B., Örnemark, U., 2014. The Fitness for Purpose of Analytical Methods – A Laboratory Guide to Method Validation and Related Topics. Eurachem Guide.

Petropoulou, S.S., Petreas, M., Park, J.S., 2016. Analytical methodology using ion-pair liquid chromatography-tandem mass spectrometry for the determination of four di-ester metabolites of organophosphate flame retardants in California human urine. J Chromatogr A 1434, 70-80.

Van den Eede, N., Maho, W., Erratico, C., Neels, H., Covaci, A., 2013a. First insights in the metabolism of phosphate flame retardants and plasticizers using human liver fractions. Toxicology Letters 223, 9-15.

Van den Eede, N., Neels, H., Jorens, P.G., Covaci, A., 2013b. Analysis of organophosphate flame retardant diester metabolites in human urine by liquid chromatography electrospray ionisation tandem mass spectrometry. J Chromatogr A 1303, 48-53.

Waters Corporation, 2021. Controlling contamination in LC/MS systems: best practices in: 715001307, D.N. (Ed.).
